# Predicting moral intelligence in nursing students and its relationships with self-compassion, and cultural competence: a cross-sectional study

**DOI:** 10.1186/s12912-022-01111-w

**Published:** 2022-11-28

**Authors:** Monir Nobahar, Sajad Yarahmadi, Nayyereh Raiesdana, Elham Shahidi Delshad, Fatemeh Hajizadegan, Farzad Ebrahimzadeh

**Affiliations:** 1 Nursing Care Research Center, Semnan University of Medical Sciences, Semnan, Iran; 2 Student Research Committee, Semnan University of Medical Sciences, Semnan, Iran; 3 Faculty of Nursing and Midwifery, Semnan University of Medical Sciences, Semnan, Iran; 4 Islamic Azad University of Borujerd, Broujerd, Iran; 5 Nutritional Health Research Center, School of Health and Nutrition, Lorestan University of Medical Sciences, Khorramabad, Iran

**Keywords:** Cultural competence, Moral intelligence, Self-compassion, Nursing students

## Abstract

**Background:**

In the recent era, nursing needs employees with moral intelligence, cultural competence, and self-compassion skills more than ever. This study aimed to determine the predictors of moral intelligence and its relationship with self-compassion and cultural competence in nursing students.

**Methods:**

This cross-sectional and multi-center descriptive study was conducted in 2022. With convenience sampling, 250 nursing students from three Iranian universities participated in this study. Data gathering included the Moral Intelligence Questionnaire, Self-Compassion Scale (short form), and Cultural Competency Questionnaire. The data were analyzed using descriptive statistics, the correlation between variables, and hierarchical regression.

**Results:**

The results showed that nursing students had good moral intelligence (72.63 ± 11.38), moderate self-compassion (37.19 ± 5.02), and poor cultural competence (50.06 ± 13.15). No statistically significant relationship was observed between self-compassion and cultural competence (*r* = 0.11, *p* = 0.07). Moral intelligence with marital status (*r* = 0.16, *p* = 0.01), academic year (*r* = 0.14, *p* = 0.03) and self-compassion (*r* = 0.33, *p* < 0.001) had a significant relationship in such a way that these variables explained 15% of moral intelligence and self-compassion had the highest impact (*p* < 0.001).

**Conclusion:**

Considering the moderate level of self-compassion and the poor level of cultural competence reported in the undergraduate nursing students, and also that self-compassion was known to be a predictive factor for moral intelligence, planners and educators must pay more attention to promoting self-compassion and cultural competency in the curriculum and conduct studies to find ways to improve them.

## Background

Moral intelligence is managing the problem-solving and conflict-resolution process with dedication and conscious participation in interpersonal and intrapersonal communication is referred to as a term that has recently been introduced in the nursing profession [1–3]. Moral intelligence can effectively make life purposeful, create meaning in life, and increase the chances of survival, prosperity, and individual productivity [2]. Applying moral intelligence can help one learn clever things and take the best course of action. It reduces risks and gives access to the best information. There is a belief that individuals with high moral intelligence always act morally [1]. Subjective moral standards, such as moral intelligence, impact nurses’ moral performance. This quality in nurses can facilitate effective communication with patients [4]. The principles of moral intelligence, such as accepting responsibility and the results of one’s behavior, having compassion for others, and forgiving oneself and others, are similar to the principles of self-compassion, and moral intelligence and self-compassion may be related to one another [1, 5].

Self-compassion is the ability to communicate with the self’s emotions and well-being [6]. There is proof that the idea of self-compassion has not gotten much attention. Self-compassion involves a desire to lessen suffering and heal oneself with kindness when one touches and opens up to sorrow, rather than ignoring or distancing oneself from it, considering one’s shortcomings as a component of the human experience and one’s failures and mistakes. Self-compassion is just as important as compassion for others because it is not only self-centered but also tends to increase feelings of compassion and concern for others. It also requires understanding one’s experience in shared human experience [7]. In fact, it implies that a person cannot be kind to others if he or she is not kind to him/herself [6]. Hagerman et al.’s study revealed that self-compassion could support academic success indicators in undergraduate nursing students while promoting compassionate care for one’s well-being, flexibility, and emotional intelligence [8]. Alquwez et al.’s study also demonstrated that nursing students’ self-compassion positively impacted their caring conduct and compassion [6].

Cultural competence is the perception of patients’ values, beliefs, and health practices that manifests itself in the ability of caregivers to communicate effectively with individuals from different cultures [9]. The caring behavior of nurses is related not only to moral issues but also to the cultural needs of patients [10]. Cultural competence in nursing is a gradual and continuous process that can provide effective, safe, and high-quality care to patients from different cultures, taking into account different aspects of their culture [11]. Providing safe, equal, and appropriate healthcare services can increase when nurses are culturally competent. This can also improve patient-nurse relationships and the monitoring and control of the disease [12]. As a result, nurses have a moral and legal need to be culturally competent to give excellent care to various patients [13]. The findings of Dur et al.’s study demonstrated that in nurses, there is a positive and significant relationship between moral intelligence and cultural sensitivity [14]. Also, Gol et al.’s study showed that nursing students’ intercultural sensitivity and cultural intelligence were positively correlated [15].

In addition to clinical qualifications, nursing students in clinical education settings should have the necessary mental and psychological traits, moral principles, and intelligence [1]. Particularly in students of professions like nursing that directly interact with people and their lives, moral intelligence plays a crucial part in the style and promotion of interpersonal connections. The nurses of tomorrow are today’s nursing students [1, 16]. By identifying strict ethical standards, they must abide by the caliber of care they deliver to patients. Increasing moral intelligence increases people’s moral responsibility and contributes to the betterment of society’s health. Although moral intelligence is crucial for nursing students, little is known about it [1]. In this regard, Poorteyimoure et al.’s study also revealed a clear and significant correlation between nursing students’ moral intelligence, clinical work experience, and professional behavior [17]. The study’s findings by Eskandari et al. have demonstrated that improving nursing students’ moral intelligence during their studies can significantly improve their work efficiency [1].

Some studies demonstrate the connection between cultural competence and morals [10, 18, 19] and even attempt to incorporate cultural competence into ethical codes [10]. According to Ho′s study in Vietnam, nursing students encounter fewer cultural challenges than nurses, so this idea has been overlooked in nursing education. However, cultural competency training is necessary to promote culturally competent care and can significantly contribute to the quality of nurses’ professional performance [20]. A systematic review also demonstrated that cultural competency training in nursing students could help cultivate knowledge, understanding, commitment, and self-confidence in providing quality care [13]. The current study aimed to identify the factors that predict moral intelligence in nursing students and their associations with self-compassion and cultural competence.

## Methods

### Design

This is a cross-sectional, multi-center descriptive study from three universities; Semnan University of Medical Sciences, Semnan, Iran; Tehran University of Medical Sciences, Tehran, Iran; and Lorestan University of Medical Sciences, Khorramabad, Iran, which was conducted from December 2021 to March 2022.

### Participants

The participants in this study were undergraduate nursing students in their second year and above. They were chosen by a convenience sampling method. The inclusion criteria for this study contained completing the first year of a nursing bachelor’s degree because, in Iran, undergraduate nursing students enter clinical environments from the second year, willingness to participate in the study, and incomplete completion of the questionnaires.

### Sample size

Based on a similar study by Gottlieb, the Pearson correlation coefficient between cultural competence and self-compassion was *r* = 0.25 [5]. The sample size was estimated based on the following formula of 164 people. Considering the design’s effect, the number was multiplied by 1/5, and with power, 90%, Z (1_a/2) =1.96, β = 0.1, a = 0.05, and Z (1_ β) =1.28, the sample size of 250 was considered satisfactory

$$\frac{{\left({Z}_{1-\alpha /2}+{Z}_{1-\beta}\right)}^2}{{\left(\frac{1}{2}\ \mathit{\ln}\frac{1+r}{1-r}\right)}^2}$$

## Instruments

Data collection tools included four questionnaires of demographic information, moral intelligence, self-compassion, and cultural competence. Permission was obtained from the instrument developers for the moral intelligence, self-compassion, and cultural competence instruments. The demographic information questionnaire included six questions: age, sex, marital status, university of study, academic year, and grade point average (Grading is based on a 0 to 20 score).

### Moral intelligence questionnaire

It was developed by Lincoln Keel with 40 items on a five-point Likert-type scale (1 = almost never, 5 = almost always). This questionnaire can measure the four dimensions of moral intelligence, including integrity (items 1–10), responsibility (items 11–20), compassion (items 21–30), and forgiveness (items 31–40). The final score is divided by two, and the obtained number is placed between the two points, 20 and 100. Finally, score categorization would be designed as 90–100 (excellent), 80–89 (very good), 70–79 (good), and 69 or lower (weak) [21]. This questionnaire was validated by Majidi et al. in Iran. Its Cronbach’s alpha coefficient is reported to be 0.88 [22].

### Self-compassion questionnaire

This tool was developed by Neff [7]. Raes created a short form of this scale with 12 items. This questionnaire has six dimensions; Self-kindness (items 2 and 6), self-blame (items 11 and 12), common human experience (items 5 and 10), isolation (items 4 and 8), mindfulness (items 3 and 7), and extreme identification (items 1 and 9). The items are rated on a five-point scale (1 = almost never, 5 = almost always). A higher score indicates a higher level of self-compassion [23]. Khanjani validated this questionnaire in Iran. The reliability of this questionnaire has been confirmed with Cronbach’s alpha coefficient of 0.86, and its reliability coefficient using the test-retest method with a one-week interval has been reported as 0.9 [24].

### Cultural competence questionnaire

This questionnaire consists of 20 items. It was developed and validated by Khanbabaei for the Iranian population. This questionnaire has three dimensions of learning and education (items 4, 8, 11, 15, and 20): awareness and knowledge (items 1, 2, 3, 7, 9) and skill (items 5, 6, 12, 13, 14, 16, 17, 18 and 19). The items are rated on a five-point scale (1 = almost never, 5 = almost always). The score categorization would be designed as 93–100 (very strong), 81-92 (strong), 80 64 (moderate), 44-63 (weak), and 20-43 (very weak). Cronbach’s alpha coefficient of this questionnaire is 0.91, and the intragroup correlation coefficient is 0.93 [9].

### Procedure Instruments

Before starting the sampling, necessary permits for data collection were obtained from the authorities. In the sampling stage, the objectives of the research were fully explained to the participants so that they could withdraw from the study at any stage if they wished. Also, they were informed that the results of the research would be provided to them if they were interested. The data was collected face to face through a paper questionnaire and with previous coordination by referring to educational and clinical environments by the researcher.

### Data analysis

Demographic variables were described using statistical indicators of absolute frequency, mean, standard deviation, skewness, and kurtosis indices. Pearson and Spearman correlation tests were used for the inferential section, and hierarchical multiple linear regression tests were used to test research hypotheses. Spearman’s test was used for ordinal variables. The maximum alpha error level to test the hypotheses was set at 0.05. Data analysis was done with SPSS 27.

## Results

Two hundred fifty nursing students from three universities participated in this study. Skewness and Kurtosis tests showed that all main variables had a normal distribution.

### Demographic information of the participants

The findings showed that the majority of the participants were female (6.59%); single (6.91%); in the age group of 21 to 23 years (6.65%); with a grade point average of 15-17 (53.2%); were studying in the second year (38%); and a majority of participants were from Semnan University of Medical Sciences (2.35%), (Table 1).

**Table 1:** Demographic characteristics and their correlation with moral intelligence in nursing students

Variables	N (%)	Moral Intelligence Mean (SD)	r (*p*-value)
Gender			−0.08 (0.21)*
Female	149 (59.60)	73.37 (9.54)	
Male	101 (40.40)	71.55 (13.58)	
Marital status			0.16 (0.01)*
Single	229 (91.60)	72.08 (11.50)	
Married	21 (8.40)	78.57 (7.96)	
Age (years)			−0.08 (0.22)**
18-20	55(22.00)	76.13 (11.54)	
21-23	164 (65.60)	71.21 (10.99)	
24-26	22 (8.80)	72.32 (11.65)	
> 26	9 (3.60)	78.22 (12.28)	
Grade Point Average (0-20)			0.06 (0.39)**
< 15	34 (13.60)	72.58 (11.09)	
15-17	133 (53.20)	71.80 (12.18)	
> 17	83 (33.20)	73.98 (10.08)	
Years of Education			−0.14 (0.03)**
Second year	95 (38.00)	75.22(9.96)	
Third year	92 (36.80)	70.34 (12.82)	
Forth year	63 (25.20)	72.07 (10.46)	
University center			0.08 (0.20)*
Semnan	88 (35.20)	72.06 (11.70)	
Tehran	83 (33.20)	71.31 (10.61)	
Lorestan	79 (31.60)	74.64 (11.64)	

*Pearson Correlation, **Spearman Correlation

### The level of dimensions in moral intelligence

Based on the results of this study, the mean and standard deviation of integrity scores was 37.29 ± 5.63, scores of responsibilities were 37.54 ± 6.08, scores of compassions were 37.41 ± 6.12, and forgiveness scores were 34.85 ± 5.79.

### Correlation between variables

The results of Pearson and Spearman correlation tests show there was a significant relationship between moral intelligence and marital status (*r* = 0.16, *p* = 0.01) and academic year (*r* = − 0.14, *p* = 0.03). So married students in a lower academic year had higher moral intelligence than others. The results of the Pearson correlation test showed that self-compassion had a significant relationship with total scores of moral intelligence (*r* = 0.33, *p* < 0.001) (Table 2). Also, the results of the Pearson correlation test showed that self-compassion had significant relationships with all dimensions of moral intelligence included; integrity (*r* = 0.31, *p* < 0.001), responsibility (*r* = 0.30, *p* < 0.001), compassion (*r* = 0.34, *p* < 0.001), and forgiveness (*r* = 0.39, *p* < 0.001).

**Table 2:** Description and correlation of moral intelligence, cultural competence, and self-compassion in nursing students

Variable (Min-Max)	Mean (SD)	Moral Intelligence r (*p*-value)*	Cultural Competence r (*p*-value)*	Self-Compassion r (*p*-value)*
Moral Intelligence (20-100)	72.63 (11.38)	1 (> 0.999)		
Cultural Competence (20-100)	50.06 (13.15)	0.01 (0.98)	1 (> 0.999)	
Self-Compassion (12-60)	37.19 (5.02)	**0.33 (< 0.001)**	0.11 (0.07)	1 (> 0.999)

* Pearson Correlation

### Hierarchical multiple linear regression predicting moral intelligence

Only the demographic variables that had a significant relationship with the dependent variable based on the correlation test were included in the regression model (Table 2). A hierarchical multiple linear regression test was performed to predict moral intelligence among the correlated variables. There was no multicollinearity between predictor variables (VIF > 2). The results of variance analysis for all the factors were statistically significant, showing the regression model’s fitness. Examining the coefficient of determination (R^2^) showed that the predictor variables could explain 15% of the changes in moral intelligence (*p* > 0.001). The findings showed that the variables of marital status (β = 0.15, *p* = 0.01), academic year (β = 0.12, *p* = 0.05) and self-compassion (β = 0.34, *p* > 0.001) can predict moral intelligence. Furthermore, the highest effect was related to self-compassion, with a coefficient of 0.34 (Table 3).

**Table 3:** Predictors of moral intelligence in nursing students

Dependent Variable	Predictive variable	B	SE	β	*P*-value	VIF	F	R^2^	*P*-value
Moral Intelligence	Marital Status	6.05	2.37	0.15	**0.01**	1.01	10.97	0.15	< 0.001*
	Years of Education	− 1.59	0.80	−0.12	**0.05**	1.01			
	Cultural Competence	−0.05	0.05	− 0.06	0.31	1.03			
	Self-Compassion	0.75	0.13	0.34	**< 0.001**	1.01			

*SE* Standard Error, β Unstandardized Coefficients, *VIF* Variance Inflation Factor, *F* value

* Multiple Regression Analyses

## Discussion

In Iranian undergraduate nursing students, the level of moral intelligence, self-compassion, and cultural competence was respectively good, moderate, and weak. The findings showed that the variables of marital status, academic year, and self-compassion could predict moral intelligence, and the most significant impact was on self-compassion. No significant relationship was seen between self-compassion and cultural competence.

The present study demonstrated that undergraduate students had a good level of moral intelligence. Mohammadi et al.’s study in Tehran, Iran, also showed a good level of moral intelligence for medical students [19]. In the study conducted by Poourtimore et al. in Urmia, Iran, nursing students had a good level of moral intelligence [17]. Moral intelligence is not intrinsic and is obtained by training and modeling. Universities are important social environments that can develop social, moral, and cultural values; moreover, they transfer advanced knowledge and capacities to students and motivate identity, emotional, behavioral, mental, and moral growth. In order to promote moral intelligence to a high level, it is necessary to include this concept in the nursing curriculum. Because nursing students, as future nurses, can improve the quality of care provided to patients and, as a result, enhance the health of society [19].

Based on this study’s results, participants’ self-compassion had a moderate level. In line with our findings, Kotera et al., in their research, reported moderate self-compassion in undergraduate nursing students in the U.K. [25]. Self-compassion is the ability to observe one’s and other’s suffering and the commitment to eliminate it [7], which is strongly related to positive mental health experiences in many student populations [26]. Self-compassion in nursing students can prepare them for compassionate care. Focusing more on this concept in nursing education allows for the growth and development of compassionate care practice [6]. The results of Chen et al.’s study in the U.S. demonstrated that self-compassion effectively reduces depression symptoms in nursing students by increasing flexibility and optimism and reducing perceived stress [27]. Therefore, educational planners must pay more attention to this concept and conduct studies to improve self-compassion in nursing students.

In our study, there was a statistically significant relationship between self-compassion and moral intelligence, which was a predictive factor for moral intelligence among undergraduate nursing students. There are some aspects of moral intelligence, like integrity (doing what we know is right and telling the truth at all times), responsibility (embracing liability for behaviors and outcomes of some behaviors and defeats), compassion (sympathetic pity and concern for the suffering or misfortune of others), and forgiveness (perception of one’s defects and wrongs) [1]. These dimensions have a close relationship with self-compassion, and achieved results in this study acknowledge all presumptions about that. Therefore, by improving self-compassion, we can expect to improve moral intelligence. Considering this result, educators could integrate self-compassion into their teaching curriculum and link it with moral intelligence.

We revealed that the cultural competence of our participants was at a weak level. The consequences of the same studies on nursing students demonstrate that cultural competence in South Korea was medium to high [12], and in Saudi Arabia was low [28]. Cultural competence is the most basic need and necessity of nursing to develop the care of patients with various backgrounds due to the increased cultural diversity and migration of nurses worldwide [20, 29]. So nurses who care for patients from various cultures should comprehend and respect several cultures and opinions. They should be competent in preparing meaningful care for patients from different cultures through effective interactions. Cultural encounters and cultural knowledge are two strong predictors of cultural competence in nursing students [27]. Since cultural knowledge can be achieved through the training of cultural competence in the curriculum of undergraduate nursing students [20], it seems necessary to provide continuous opportunities to improve the cultural competence of nursing students so that they interact more with different cultures.

Although, we did not observe a significant relationship between cultural competence and moral intelligence in undergraduate nursing students. However, the perception and intuition of nurses about the vulnerability of patients from various cultures illustrate their moral sensitivities in conducting ethical decisions [10]. Due to the lack of a similar study in this field, it seems necessary to conduct more studies.

Moreover, according to the study’s results, a significant relationship was not seen between self-compassion and cultural competence in undergraduate nursing students. Gottlieb’s study in the U.S. demonstrated a significant relationship between self-compassion and cultural competence in social work students, and self-compassion was considered a strong predictor of cultural competence in these students [5]. Acquiring cultural competence leads to acceptance of oneself and others and the promotion of compassionate care, so it is necessary to conduct more research on cultural competence in the community of nursing students [5].

This study showed that married students had higher moral intelligence than single students. Also, Gholami et al.’s study indicated that married nurses had higher moral intelligence. Since different genetic, psychological, and social factors affect moral intelligence [4], it seems that high moral intelligence in married students is more due to having social life skills in married people.

This study showed that students in higher academic years have lower moral intelligence. Among the possible reasons, we can mention the choice of wrong models during education, the student’s attention to improving practical skills, and neglecting morals. Teaching the basic nursing courses and ethics in the undergraduate nursing curriculum in the first years of study influences the fading of moral intelligence during the study course. We could find no accurate studies that addressed this issue.

Finally, the results of this research can be used to develop knowledge related to the concept of moral intelligence, which has recently entered the nursing profession and identify ways to improve it. The results of this study can help policy makers, nursing managers, and educational designers to design and implement more effective educational programs in order to have nursing students and nurses with high moral intelligence.

One of the strengths of the present study is examining and measuring the correlation of several concepts with moral intelligence. The second strength of this study is the multi-center study design that was carried out on students in medical sciences universities in three provinces that can develop the generalizability of the outcomes. There were also limitations in this study. First, there were many questionnaires, and participants completed them slowly, so it was challenging to convince them. We overcame this problem more patiently and continued communication with the research participants. Since the data gathering tools were completed self-reporting, the respondents’ bias and social desirability can be increased. On the other hand, convenience sampling limited the results’ generalizability. Moreover, understanding the causality of the relationships between variables requires further studies. Therefore, some observational studies are needed to clarify the relationships between variables.

## Conclusion

The results of this study demonstrated that the moral intelligence of the Iranian undergraduate nursing students was at a reasonable level, and there was a relationship between moral intelligence and marital status, students’ academic year, and self-compassion. Also, married students in the lower academic year had higher moral intelligence than others. According to the fact that the level of self-compassion was reported as moderate and the level of cultural competency was reported to be weak, and also self-compassion was found to be a predictive factor for moral intelligence, planners and educators must pay more attention to the promotion of self-compassion and cultural competence in curricula and studies are conducted to find ways to improve them.

## Data Availability

The datasets generated and analyzed during the current study are not publicly available due to privacy protection and ethical considerations but are available from the corresponding author upon reasonable request.
